# Home Fire Safety Visits by the Fire and Rescue Service: a qualitative study of the perspectives of firefighters, advocates and service leaders

**DOI:** 10.1186/s12889-025-24429-x

**Published:** 2025-09-30

**Authors:** Sarah Hampton, Helen Anderson, Arabella Scantlebury, Joy Adamson

**Affiliations:** 1 Department of Clinical, Educational and Health Psychology, University College London, London, WC1E 7HB UK; 2 Department of Health Sciences, University of York, York, YO10 5NB UK; 3 Centre for Evidence and Implementation Science, College of Social Science, University of Birmingham, Birmingham, B15 2TT UK

**Keywords:** Fire and Rescue Service, Older patients, Integrated care

## Abstract

**Background:**

The UK Fire and Rescue Service (FRS) routinely deliver Home Fire Safety Visits (HFSVs) in people’s homes. HFSVs offer support with fire safety and a range of health-related issues. This study aimed to explore the perspectives of those delivering and designing the HFSV service.

**Methods:**

Twenty eight members of the FRS who deliver HFSVs and service leaders involved in HFSV service-design were interviewed. Data were analysed thematically.

**Results:**

Participants described a cultural shift within the FRS from response to prevention and public health work. Most felt positively about this change, though some reported difficulty adjusting to their new role. Working with other services was seen as integral to the HFSV service due to the links between fire risk and other facets of health. However, participants felt the FRS were expected to plug gaps in other services, despite not always feeling equipped to do so. Challenges were identified in reaching and supporting underserved groups (e.g. mental health issues and dementia).

**Conclusions:**

HFSVs could address a range of health-related needs. However, whether the FRS should be expected to fill gaps in other services needs further exploration. Supporting underserved groups via HFSVs is important and warrants further investigation.

## Introduction

The number of home fires has substantially reduced in recent decades, in part due to the efficacy of prevention work [1, 2]. This reduction in fires has increased capacity within the UK FRS and resulted in a significant cultural change in recent years [3].

The National Health and Social Care Service (NHS), is increasingly under pressure and financial strain [4] and has been less effective in engaging with prevention than the FRS [5]. The FRS has expanded its role into public health in an attempt to ease the burden on the NHS. The diversification of the FRS fits alongside a broader trend towards employing those outside the health sector to fill gaps in public health [6, 7].

The FRS have traditionally delivered Home Fire Safety Visits (HFSVs) in people’s homes to fit smoke alarms and other fire safety equipment, and provide advice on fire prevention. Approximately 670,000 visits are carried out each year [8]. In 2015 Public Health England, the Chief Fire Officers Association, NHS England, the Local Government Association and Age UK issued a consensus statement [9], to work together to ensure people with complex needs received integrated personalised care. As a result, many FRS expanded their HFSV to include an assessment of risk factors for falls, smoking cessation, fuel poverty, social isolation, and other factors. HFSVs are delivered by firefighters and prevention advocates (also known by various other names such as prevention advisors or safe and well officers, hereafter referred to as ‘advocates’). Advocates are a non-operational role within the FRS focusing on fire prevention and community work. Service users are recruited in various ways including self-referrals, referrals by other services, and cold calls. There are striking inequalities in who tends to be affected by home fires [10] and as such the HFSV service aims to target those most vulnerable to fire, including older people, those with substance dependency, and those with poor mobility. The HFSV service may improve health outcomes and quality of life, particularly for those facing health-related inequalities.

While there is little existing data into the effectiveness of HFSVs, there is tentative evidence that service users find the visits useful and report making changes to their behaviour following a visit [11]. Evidence exploring FRS members’ perceptions of the HFSVs and their changing professional identity is limited. Two small qualitative studies have explored FRS member perspectives on their role, including HFSVs [12, 13]. This qualitative study, conducted alongside the UK’s largest FRS trial (FIREFLI), builds on this limited evidence base by including service leader perspectives. This exploratory qualitative study aimed to explore experiences and acceptability of the HFSVs within an evolving FRS, from the perspective of those delivering HFSVs and service leaders involved in the design of the HFSV service.

## Methods

### Study design

This qualitative study was conducted alongside a randomised controlled trial, the ‘Do Safe and Well Visits delivered by the Fire and Rescue Service reduce falls and improve quality of life among older people (FIREFLI)’ study. FIREFLI aimed to assess the effectiveness of HFSVs in reducing falls and improving quality of life, alongside other outcomes, among older adults. The trial is described in more detail elsewhere [14]. FIREFLI received ethical approval from the West Midlands - Coventry & Warwickshire Research Ethics Committee (reference number 21/WM/0050).

### Sampling and recruitment

Semi-structured interviews were conducted with firefighters and advocates who deliver HFSVs and service leaders involved in the design and implementation of HFSVs. Participants were from two FRS, one in the north and one in the south of England. Participants were recruited through key contacts at the participating Fire and Rescue Services. Variation in service provider roles (i.e. both firefighters and advocates) was sought.

### Data collection

Topics for both service provider and leader interviews included barriers and facilitators to delivering the HFSVs and integration of the HFSV service with health and social care services. Topic guides were developed from relevant intervention fidelity literature [15] and study aims focussed towards understanding experiences of delivering HFSV from multiple stakeholder perspectives. Interviews were conducted remotely, were audio recorded and transcribed. All interviewees gave written informed consent.

### Data analysis

This study was an applied piece of health and care research, which was atheoretical. We chose to apply [16] definition of thematic analysis to our data, a method which is widely used in applied health and social care research and is renowned for its flexibility and being unbound by particular theoretical commitments. We adopted a descriptive rather than interpretive approach to thematic analysis, which was focussed on providing an in-depth, but pragmatic understanding of how HFSVs are being delivered in two FRS in England. Following familiarisation, initial codes were developed by coding the transcripts line-by-line. Coding was conducted largely deductively, guided by the research questions, though inductive codes and patterns within the data were sought wherever possible. Codes were then grouped into mid-level subthemes and final-level themes. Themes and subthemes were checked for internal coherence and lack of overlap by removing, splitting or combining. The first author took the lead on analysis at each stage, discussing findings with the other authors. All authors are experienced applied qualitative health and social care researchers and one author is a registered nurse (HA). All authors have no previous experience with the FRS and this may have influenced how they engaged with the data. FRS leaders at participating services were part of the wider FIREFLI trial team and provided the authors with context for the HFSV service and insights into the functioning of the FRS.

## Results

17 service providers (6 firefighters, 11 advocates) and 11 service leaders were interviewed. Service leaders included senior members of the FRS (both those directly responsible for the HFSV service and those with a lighter level overview; *n* = 7) and healthcare services (including physiotherapy,community geriatrics and falls prevention services; *n* = 4). Service providers had an average of 13 years of experience within the FRS (range = 3 months-25 years).

### Culture change within the FRS—shifting towards fire prevention and a more holistic approach to health and wellbeing

Participants emphasised that the HFSV service has expanded over recent years from fire safety to a more holistic approach to health and wellbeing. While service leaders and providers tended to view fire safety support as the purpose of HFSVs, they also recognised the value of supporting broader wellbeing. Participants recognised a need for HFSVs to be tailored to the individual’s needs, and felt that the expanding remit of the HFSVs facilitated this individualised approach. This view was common across experience levels. One experienced firefighter stated,

*‘Years ago it was literally*,* right we’re the Fire Service*,* we’ve come to make sure you’re fire safe and that was it. But now*,* it’s everything. It’s more a social call than anything because we pick up on everything no matter what it is. […] I think we’re one step away from being social workers to be fair.’ (Firefighter)*

Participants alluded to resistance among firefighters to the cultural shifts within the FRS, and it was felt that firefighters’ expectations and the reality of their role were not always in alignment. Service leaders sometimes felt that the shift towards topics beyond fire safety had met with greater resistance than the shift from fire response to prevention. This resistance was considered more prevalent amongst the older generation of firefighters, who were perceived as less willing to adapt. Interestingly, this hesitancy also extended to service providers, who despite being generally in favour of the widening FRS remit described initial trepidation when their role began to change. This was especially true for those with more years of experience in the role, with one experienced firefighter stating,

*‘My initial reaction was*,* we’re not care workers. We’re not social workers. But […] I’m big pusher for it now*,* especially because I look at it with the other shoe. If it was my parents how would I want them to be treated and that’s how I kind of do all my jobs*,* is how would I want my parents and my family to be treated.’ (Firefighter)*

Service providers and leaders felt that the advocate role supported the expansion of the HFSV service’s remit - advocates support the HFSV service by taking on more complex cases. While advocates generally felt well supported in their role, a minority desired more support with the difficult situations they encounter and could sometimes feel frustrated with the limits of their role. One experienced advocate who had observed changes in the role over the years commented,

‘*I think the people at the top who make the decisions don’t realise how much the job has developed. So it’s not really reflected in the support that we get. It’s not seen as an operational role and it’s very much an operational role. We’re a frontline service*.’ (Advocate)

### An integrated approach to prevention - FRS as facilitators of integrated health and social care

Partnership working was integral to the HFSV service. For instance, the FRS described jointly providing HFSVs and providing and receiving training from other services. More broadly, the FRS were considered, through HFSVs, to facilitate an integrated approach to care. A practical example of this approach was the creation of a falls team in association with the HFSV service at one of the sites. As part of this team, advocates respond to older individuals who have had a non-urgent fall and carry out observations, refer to other services, and offer a HFSV if appropriate.

*‘I think it’s a fantastic service that we deliver because the person is also kept safe from fire*,* so they’re only been picked up within an hour*,* which that’s very unusual if you call an ambulance they’re a lot longer and the longer lies cause complications […] We’re taking their observations so they’re getting a mini kind of health check aren’t they with their observations and then we keep them safe from fire because we give them a full safe and well visit.’ (Service leader)*

The FRS was thought to be particularly well placed to provide a link between services, given that their status as a well-respected service allows them to reach individuals who may not be known to other services. The FRS was commonly felt to be perceived as a trusted service in comparison to other services, and this was felt to make service users more receptive to the HFSVs.

‘*Fire & Rescue Services do rank very highly in terms of being able to access communities and seen as a non-judgemental service. Obviously they’ve got a better reach than the police because that’s seen as a punitive service and increasingly they’ve got a better reach than Health & Social Care which is seen as a judgemental service*.’ (Service leader)

### Reaching and supporting vulnerable people

Older people and those experiencing hoarding, domestic violence, addiction and mental health issues were regularly identified as an underserved group and a number of challenges in identifying, reaching, and supporting these individuals as part of the HFSV were provided. It was felt that this group could be reluctant for officers to enter their home due to feeling ashamed. Repeat visits and working with other agencies, such as housing, were identified as potential solutions to this challenge, *‘it’s kind of working with that person… we try and involve other agencies and that’s how we go about trying to get in through the door.’* (Service leader). Generally, service providers were content with their training, however a training need was identified around supporting people with mental health issues who were consistently described as “*the most tricky ones to get through to*”. Despite this, reaching underserved groups remains a challenge, with those experiencing hoarding, addiction and other issues considered to be particularly resistant to behaviour change.

*‘I’m not going to walk into a house and tell an alcoholic that if you stop drinking and stop smoking*,* you wouldn’t die in a fire. I don’t realistically think that that’s ever going to be enough for that person to change those*,* you know*,* types of behaviour*,* those extreme behaviours. I can refer them and sign-post them on to other agencies and other services that can support them with that.’ (Advocate)*

HFSVs often target an older population, and cognitive issues such as dementia could pose a barrier to individuals understanding and retaining the information given during the visits. Working with family members/carers was sometimes helpful for overcoming these issues. Language and cultural barriers were also prevalent. Participants tried to overcome these issues by leaving leaflets in different languages or by working with interpreters and family members who speak English. Reaching those of other cultures could also be challenging, and service leaders and providers identified a need to reach out to minority communities.

‘*cultural and language barriers are our biggest ones but we do try quite a lot. We go into mosques. We go into areas where these cultures kind… we’ve got a chicken factory and I think 90% of the staff there are kind of Lithuanian*,* so we’ll go into there and try and engage with people on their lunch breaks and things like that*.’ (Advocate)

### Challenges associated with working with other services

Service providers talked of making and receiving referrals to health and social care services (including housing, smoking cessation services, deaf services etc.). While most participants felt there were smooth channels of inter-agency communication, services often had their own unique referral pathways and this could create inefficiencies across the system. Service providers further spoke of being expected to take on large amounts of referrals for other services, ‘*We’re kind of the “go to” people*,* we’re the experts for doing the referrals*’. As a result, the FRS were described as “plugging gaps in other services.” However, whether the FRS were equipped to perform this role, was brought into question with the health and social care system considered to have different priorities to FRS. One health service leader working in falls, for example, spoke of difficulties receiving appropriate referrals from the FRS. A lack of FRS expertise in health-related referrals appeared to lead to overly inclusive referrals. This issue was ultimately resolved by introducing an intermediate social prescriber service to bridge the gaps between the FRS and health, resulting in more appropriate referrals.

*‘We found there was a huge gulf basically between what [the FRS] were able to do and what we needed to do and what it actually initially did was just create this tsunami of referrals into our service.’ (Service leader)*

Service providers sometimes expressed concern that other services could inappropriately use the FRS as a lever for their goals, including settling disputes between services.

‘*Children’s Services have got concern*,* so they want the Fire Service to come. But they just want us to put down the things that they already know and then they can use it for kind of enforcement and things like that. So if we say there’s a fire risk there and Social Services can add it to a list to get the children taken away.*’ (Advocate)

While it was acknowledged that working with other services allows the FRS to identify those most in need of the HFSVs, service providers sometimes felt the need to distance themselves from other, less trusted, services as service users could be less receptive if they perceived the visits to be associated with these services.

‘*if [the referral has] come from Social Services or the police*,* I think they think we’re spying for them and*,* do you know what I mean*,* so we get put on that level as well where they’re a bit wary of us when we go in and maybe don’t want to be honest with us*.’ (Advocate)

## Discussion

This study explored FRS service providers’ and leaders’ perspectives on HFSVs. Participants saw HFSVs as part of a cultural shift from response to prevention and public health work. This cultural shift was generally welcomed, though participants alluded to some firefighters struggling to adapt to their changing role. Participants felt that working with other services could provide opportunities to improve service users’ wellbeing, though the FRS could sometimes be expected to plug gaps in other services in inappropriate ways. Reaching and supporting vulnerable groups was identified as a key challenge for the HFSV service, and the need to build trust with vulnerable service users was highlighted.

In recent years, the FRS have expanded their remit to include public health, including the delivery of HFSVs. There is some small-scale qualitative evidence to suggest that FRS members are broadly open to supporting health within the community. However, FRS members can sometimes feel a misalignment between their duties and what they perceive to be their core role, and do not always feel sufficiently trained to deliver their expanded role [12, 13]. This study is one of the first to explore firefighters’ perceptions of their changing professional role, and the first to seek the views of both service providers and leaders. Similar to [12], we report mixed views from service providers, with some expressing enthusiasm for the benefits of the FRS moving into public health, and others expressing a mismatch between the expectations and realities of firefighters’ roles. In our sample, negative views tended to be attributed to the older generation of firefighters. More generally, the service provider views we report are more positive than those previously reported. This may be due to the larger number of advocates that made up our service provider sample, or may reflect changing attitudes towards HFSVs as they have become more established during recent years.

Our findings suggest that the FRS may be well placed to offer a holistic service such as the HFSVs, especially to underserved groups who may be less receptive to other services, given the status of the FRS as a trusted service. However, participants also spoke of the challenges of reaching underserved groups, such as those with mental health issues, who can mistrust the FRS and be unreceptive to HFSVs. Given striking inequalities in fire and health related vulnerabilities, supporting underserved groups via HFSVs is an important issue that warrants further exploration and resources. Indeed, the findings add to a small but growing body of evidence [12, 13] that some service providers desire more extensive training in areas beyond fire safety, and that training in mental health is an area of particular need.

Furthermore, this study highlights barriers to the FRS working with other services to facilitate an integrated approach to prevention. Expecting the FRS to plug gaps in other services, such as assisting in child protection matters and taking on large numbers of referrals, may be inappropriate as the FRS may not be equipped to fulfil these roles. The extent to which the FRS should be expected to fill gaps in other services is an issue that needs further exploration.

### Limitations

All interviewees were from two Fire and Rescue services and so may not be representative of the views of all FRS services and/or personnel across the UK. Interviewees included a relatively small number of firefighters and non-FRS service leaders, and service users’ perspectives are not presented and should be explored in future research.

## Conclusions

Given the increased capacity of the FRS in recent decades, and the links between fire safety and health, the FRS may be well placed to provide public health advice and signposting via SWVs. This expansion of the FRS’s role may help ease the burden of the increasingly stretched NHS. Furthermore, the reputation of the FRS as a trustworthy, non-judgemental service may help the FRS to reach and support those unknown or unreceptive to other services. However, the FRS may sometimes be requested to perform duties beyond their role or skillset. The extent to which the FRS should be expected to fill gaps in other health and social care services is therefore an issue that needs further exploration. Supporting underserved groups via SWVs can be challenging, and this important issue warrants further exploration and resources.

## Data Availability

The datasets generated during the current study are not publicly available in order to respect the privacy of individuals that participated in the study but are available from the corresponding author on reasonable request.

## Abbreviations

FRS: Fire and Rescue Service
HFSV: Home Fire Safety Visit
NHS: National Health and Social Care Service
